# Clonal relatedness of carbapenem-resistant *Acinetobacter baumannii*: high prevalence of ST136^pas^ in a burn center

**DOI:** 10.1186/s12941-023-00589-9

**Published:** 2023-05-06

**Authors:** Farzaneh Firoozeh, Mahnaz Nikibakhsh, Farzad Badmasti, Mohammad Zibaei, Vajihe Sadat Nikbin

**Affiliations:** 1 Department of Microbiology, School of Medicine, Alborz University of Medical Sciences, Karaj, Iran; 2 Evidence-based Phytotherapy and Complementary Medicine Research Center, Alborz University of Medical Sciences, Karaj, Iran; 3 Department of Bacteriology, Pasteur Institute of Iran, Tehran, Iran; 4 Department of Parasitology and Mycology, School of Medicine, Alborz University of Medical Sciences, Karaj, Iran

**Keywords:** Carbapenem resistant *A. baumannii*, *bla*_OXA−51−_like allele, *amp*C allele, Clonal complex, Burn patient

## Abstract

**Background:**

Carbapenem-resistant *Acinetobacter baumannii* (CRAB) is a global health crisis. This study aimed to determine the clonal relatedness of antibiotic-resistant *A. baumannii* isolates in hospitalized patients who suffered from burn wound infection.

**Methods:**

One hundred and six *A. baumannii* isolates from 562 patients with burn wound infections, were identified and examined for antimicrobial susceptibility. Detection and characterization of carbapenem-hydrolyzing class D OXA-type beta-lactamases (CHDLs) were performed by PCR assays. The clonal relatedness of *A. baumannii* isolates was determined by multilocus sequence typing (MLST) according to the Pasteur scheme, dual-sequence typing of *bla*_OXA−51_-like and *ampC* genes, and RAPD-PCR method.

**Results:**

All isolates were carbapenem-resistant while susceptible to colistin, minocycline, doxycycline, and ampicillin-sulbactam. The intrinsic *bla*_OXA−51_-like was detected in all isolates, and *bla*_OXA−23_-like was identified in 92.5% of isolates. However, *bla*_OXA−143_-like and *bla*_OXA−58_-like genes were not detected among isolates. Four distinct *bla*_OXA−51_-like alleles were determined as follows: *bla*_OXA−317_ (67.0%), *bla*_OXA−90_ (9.4%), *bla*_OXA−69_ (17.0%), and *bla*_OXA−64_ (6.6%) and four *ampC* (*bla*_ADC_) allele types including *ampC-25* (6.6%), *ampC-39* (9.4%), *ampC-1* (17.0%), and *bla*_ADC−88_ (67.0%) were identified. MLST (Pasteur scheme) analysis revealed four ST types including ST136 (singleton), ST1 (CC1), ST25 (CC25), and ST78 (singleton) in 71, 18, 7, and 10 of *A. baumannii* strains, respectively. Five RAPD clusters including A (1.9%), B (26.4%), C (57.5%), D (7.5%), and E (1.9%) were characterized and 5 (4.7%) strains were found to be singletons.

**Conclusion:**

The present study demonstrated that there was a high prevalence of *bla*_OXA−23_-like producing CRAB in the clinical setting. The majority of isolates belonged to ST136 (singleton). However, *bla*_OXA−23_-like producing multi-drug resistant international clones including ST1, and emerging lineages (e.g. ST25 and ST78) were also identified. Interestingly, in this study ST2 was not detected.

## Introduction

Burn patients are at high risk for nosocomial infections during hospitalization [1]. *Acinetobacter baumannii* has now emerged as one of the most important hospital-acquired pathogens in burn patients [2]. *A. baumannii* is not only involved in wound infection in burn patients but also leads to infections such as bacteremia, pneumonia and urinary tract infections in these patients [3]. This bacterium has also become an increasing threat to hospitalized patients due to a lack of response to treatment as a result of the rapid acquisition of multiple antibiotic resistance elements [4]. According to CDC reports, carbapenem-resistant *Acinetobacter* strains have been ranked as an urgent threat which also have multiple simultaneous resistance to other antibiotics [5]. Resistance to carbapenems is commonly associated with carbapenem-hydrolyzing class D beta-lactamases (CHDLs), known as OXA-type carbapenemases enzymes [6]. Based on studies, six sub-classes of CHDLs, including intrinsic chromosomal OXA-51-like enzymes and acquired OXA-23-like, OXA-24-like, OXA-58-like, OXA-143-like and OXA-235-like, have been identified in *A. baumannii* [7].

The OXA-23 is the first enzyme described in this group, which also has a global distribution and appears in international clonal lineages I (CC1) or II (CC2) [8]. According to the available evidence from molecular epidemiology studies, the structural population of *A. baumannii* consists of nine distinct clonal lineages, among them the international clones I and II have global expansion and international clone III (CC3) has been reported from many countries [9]. The increasing trend of resistance needs rigorous epidemiological studies of *A. baumannii* strains using various molecular typing methods, including multilocus sequence typing (MLST) as a reference and standard approach [9, 10]. However, this method is quite expensive and time-consuming [11, 12]. Therefore, a new, reliable, rapid and cost-effective method is needed. It has been demonstrated that sequence-based typing of both *bla*_OXA−51_-like and *ampC* is a discriminative and reliable method that can distinguish *A. baumannii* isolates at the clonal complex level [13]. Given this background and the importance of *A. baumannii* infections, this study aimed to determine the clonal relatedness of antibiotic resistant *A. baumannii* isolates in hospitalized patients with burn wound infection.

## Methods and materials

### Sample collection and identification of ***A. baumannii*** isolates

A total of 106 non-duplicated *A. baumannii* isolates were collected from hospitalized burn patients at Shahid Motahari Burn Center in Tehran, Iran from February 2020 to March 2021. All isolates were phenotypically identified by biochemical and standard microbiological methods [14]. Genetic confirmation of isolates as *A. baumannii* was performed by PCR amplification and sequencing of the *rpo*B gene [15].

### Antibiotic susceptibility profile

For determination of antibiotic susceptibility profile, the Kirby-Bauer disk diffusion susceptibility test was done. The antibiotic disks used were imipenem (IMP), doxycycline (DXT), minocycline (MN), ciprofloxacin (CIP), gentamicin (GM), trimethoprim/sulfamethoxazole (SXT), ampicillin-sulbactam (SAM), ceftazidime (CAZ) (Mast Group Ltd, UK). The minimum inhibitory concentration (MICs) values of colistin were determined using the broth microdilution method. The performance for antibiotic susceptibility testing complied with the CLSI standard guidelines [16]. The quality control strains were *Escherichia coli* ATCC 25922 and *Pseudomonas aeruginosa* ATCC 27853. Based on the antibiotic resistance profile, the *A. baumannii* strains were categorized as MDR, XDR, or PDR using Magiorakos et al. criteria [17].

### Detection and characterization of OXA-type carbapenemases genes

For this purpose, genomic DNA was extracted from all isolates by boiling method and stored at − 20 °C as a DNA template for PCR assays. The presence of OXA-type carbapenemase genes including the genes encoding OXA-23-like, OXA-58-like, OXA-51-like, and OXA-143-like was investigated by PCR as previously described [18, 19].

### Allele typing of *bla*_OXA−51_-like and *ampC*

Sequence-based typing (SBT) of *bla*_OXA−51_-like and *ampC* genes was performed by PCR sequencing. The primers and PCR conditions used to amplify targeted genes were described previously [21]. The purified DNA products were sequenced by Bioneer Company (Seoul, Korea) using the ABI 3730xl DNA analyzer (Life Technologies). The CLC Main workbench software version 5.5 (CLCBio, Aarhus, Denmark) was used for data analyses. The DNA sequences were compared against the GenBank database (https://www.ncbi.nlm.nih.gov/genbank/) using BLASTn tool and the allele number of each gene was identified.

### Random amplified polymorphic DNA (RAPD)

The genetic relatedness of *A. baumannii* isolates was determined by random amplified polymorphic DNA (RAPD) analysis using DAF4 primer as described by Grundmann et al. [20]. RAPD-PCR products were run in an agarose gel and analysis of DNA bands was performed by the unweighted pair-group method with arithmetic averages (UPGMA) clustering using GelCompare II software version 5.10 (Applied Maths, Sint-Martens-Latem, Belgium). A similarity cut-off of 80% was considered for clustering the isolates in the same genotype.

### Multilocus sequence typing

Multilocus sequence typing was performed to determine sequence types (STs) according to the Pasteur scheme [21]. In this regard, internal fragments of the seven housekeeping genes, including *gltA, pyrG, fusA, recA, cpn60, rplB* and *rpoB*, were amplified according to the conditions registered in http://pubmlst.org/abaumannii/. After DNA amplification, sequencing was performed using the ABI 3730xl DNA Analyzer (Life Technologies), and the obtained nucleotide sequences were compared to the known alleles in the *A. baumannii* PubMLST database to find the exact locus number. The eBURST analysis was applied for MLST data using PHYLOViZ software [22], and sequence types (STs) of strains were identified by seven locus numbers. Clonal relatedness of ST136 with other sequence types was depicted using this tool. The criterion for belonging to the same clonal complex was the sharing of four to seven alleles [3].

## Results

### Identification of *A. baumannii* isolates

In this cross-sectional study, 106 non-duplicated *A. baumannii* were isolated from burned wound samples of the 106 hospitalized patients (28.3% female and 71.7% male). Burn wound infections were diagnosed based on the presence of cellulitis and high concentrations (>10^5^ organisms/g of tissue) of bacteria in the burn wound [23]. The patients had a mean age of 32.4 years (range: 11 months to 84 years). Burns caused by gas were the most frequent (26.4%), followed by open flame (16.9%), gasoline (15.1%), scald (15.1%), electricity (12.3%), acid (3.8%), and other flammable materials (10.4%). See Table 1. The overall mortality rate was 21.7%.

**Table 1:** Information on burn patients demographic

Characteristic	Male, N (%)	Female, N (%)	Total, N (%)
**Age (years)**			
≤ 10	8 (10.5)	11 (36.7)	19 (17.9)
10–25	17 (22.4)	5 (16.7)	22 (20.8)
26–50	37 (48.7)	10 (33.3.)	47(44.3)
51–84	14 (18.4)	4 (13.3)	18 (17.0)
Mean ± SD			21.5 ± 9.1
**Cause of burn**			
Gas	24 (31.6)	4 (13.3)	28 (26.4)
Gasoline	13 (17.1)	3 (10.0)	16. (15.1)
Scald	6 (7.9)	10 (33.3)	16 (15.1)
Electricity	13 (17.1)	0 (0.0)	13 (12.3)
Flame	13 (17.1)	5 (16.7)	18 (16.9)
Acid	2 (2.6)	2 (6.7)	4 (3.8)
Other flammable materials	5 (6.6)	6 (20.0)	11 (10.4)
**Burn surface area (%)**			
˂ 15%	12 (15.8)	3 (10.0)	15 (14.2)
15–25%	18 (23.7)	8 (26.7)	26 (24.5)
26–50%	32 (42.1)	14 (46.6)	46 (43.4)
> 50%	14 (18.4)	5 (16.7)	19 (17.9)
Mean ± SD			26.5 ± 11.9
**Length of hospital stay (days)**			
˂ 15	32 (42.1)	10 (33.3)	42 (39.6)
15–30	34 (44.7)	17 (56.7)	51 (48.1)
> 30	10 (13.2)	3 (10.0)	13(12.3)
Mean ± SD			35.3 ± 16.2
**Mortality**			
Yes	19 (25.0)	4 (13.3)	23 (21.7)
No	57 (75.0)	26 (86.7)	83 (78.3)
**Total**	76 (71.7)	30 (28.3)	

### Antimicrobial resistance

The highest amount of antibiotic resistance was seen in imipenem, ceftazidime, trimethoprim/sulfamethoxazole, ciprofloxacin, and gentamicin. On the other hand, isolates were susceptible to colistin, minocycline, doxycycline and ampicillin-sulbactam with sensitivity rates of 100%, 96.2%, 95.3%, and 94.3%, respectively. Based on Magiorakos et al. criteria, all isolates were absolutely categorized as MDR strains [17].

### Detection and characterization of OXA-type carbapenemases genes

Findings from the detection of OXA-carbapenemase genes showed that all isolates were positive for *bla*_OXA−51_-like, and 98 (92.5%) of the isolates harbored *bla*_OXA−23_-like gene. In addition, all isolates were negative for *bla*_OXA−143_-like and *bla*_OXA−58_-like genes.

### Results of allele typing of *bla*_*OXA−51*_-like and *ampC*

Sequence-based typing (SBT) data highlighted that there were four distinct *bla*_OXA−51_-like alleles as follows: *bla*_OXA−317_ (71/106, 67.0%), *bla*_OXA−90_ (10/106, 9.4%), *bla*_OXA−69_ (18/106, 17.0%), and *bla*_OXA−64_ (7/106, 6.6%). Moreover, SBT of the *ampC* (*bla*_ADC_) confirmed four allele types including *ampC-25* (7/106, 6.6%), *ampC-39* (10/106, 9.4%), *ampC-1* (18/106, 17.0%) and *bla*_ADC−88_ (71/106, 67.0%). See Table 2. The clonal complexes (CCs) of *A. baumannii* strains were determined according to previous reports [11]. Furthermore, *bla*_OXA−69_/*ampC-1* and *bla*_OXA−64_/*ampC-25* belonged to CC1, CC25, respectively. Whereas *bla*_OXA−317_/*bla*_ADC−88_ and *bla*_OXA−90_/*ampC-39* were identified as singletons.

**Table 2:** Antimicrobial susceptibility profiles, OXA-type carbapenemases genes, *bla*_OXA−51_-like and *ampC* allele types, MLST sequence types, clonal complexes and RAPD-PCR clusters of 106 carbapenem-resistant *A. baumannii* isolates

Resistance pattern (Disk diffusion)	Isolate (N)	MIC colistin (µg/ml)	*bla*_OXA−23_-like	*bla*_OXA−51_-like allele	*ampC* (*bla*_ADC_) allele number	Clonal complex	MLST sequence type (Pasteur scheme)	RAPD cluster
IMP, CAZ, SXT	1	0.75	+	*bla*_OXA−69_	*ampC-1*	CC1	ST1	D
	1	0.75	+	*bla*_OXA−317_	*bla*_ADC−88_	Singleton	ST136	C
	1	0.75	+	*bla*_OXA−317_	*bla*_ADC−88_	Singleton	ST136	Singleton
	1	0.75	-	*bla*_OXA−69_	*ampC-1*	CC1	ST1	C
	1	0.75	-	*bla*_OXA−64_	*ampC-25*	CC25	ST25	Singleton
IMP, CAZ, SXT, GM, CIP	3	0.75	+	*bla*_OXA−317_	*bla*_ADC−88_	Singleton	ST136	C
	2	0.75	+	*bla*_OXA−69_	*ampC-1*	CC1	ST1	B
	2	0.75	+	*bla*_OXA−69_	*ampC-1*	CC1	ST1	C
	2	0.75	+	*bla*_OXA−317_	*bla*_ADC−88_	Singleton	ST136	B
	1	0.75	+	*bla*_OXA−317_	*bla*_ADC−88_	Singleton	ST136	Singleton
	23	1	+	*bla*_OXA−317_	*bla*_ADC−88_	Singleton	ST136	C
	8	1	+	*bla*_OXA−317_	*bla*_ADC−88_	Singleton	ST136	B
	4	1	+	*bla*_OXA−90_	*ampC-39*	Singleton	ST78	C
	3	1	+	*bla*_OXA−317_	*bla*_ADC−88_	Singleton	ST136	D
	3	1	+	*bla*_OXA−64_	*ampC-25*	CC25	ST25	C
	2	1	+	*bla*_OXA−69_	*ampC-1*	CC1	ST1	D
	2	1	+	*bla*_OXA−69_	*ampC-1*	CC1	ST1	B
	2	1	+	*bla*_OXA−90_	*ampC-39*	Singleton	ST78	B
	1	1	+	*bla*_OXA−69_	*ampC-1*	CC1	ST1	E
	1	1	+	*bla*_OXA−69_	*ampC-1*	CC1	ST1	Singleton
	1	1	+	*bla*_OXA−69_	*ampC-1*	CC1	ST1	C
	1	1	+	*bla*_OXA−64_	*ampC-25*	CC25	ST25	B
	1	1	-	*bla*_OXA−69_	*ampC-1*	CC1	ST1	E
	1	1	-	*bla*_OXA−69_	*ampC-1*	CC1	ST1	C
	1	1	-	*bla*_OXA−317_	*bla*_ADC−88_	Singleton	ST136	C
	1	1	+	*bla*_OXA−90_	*ampC-39*	Singleton	ST78	A
	1	1	+	*bla*_OXA−317_	*bla*_ADC−88_	Singleton	ST136	C
	1	1	+	*bla*_OXA−317_	*bla*_ADC−88_	Singleton	ST136	B
	1	1	+	*bla*_OXA−317_	*bla*_ADC−88_	Singleton	ST136	D
	1	1	+	*bla*_OXA−317_	*bla*_ADC−88_	Singleton	ST136	A
	1	1	+	*bla*_OXA−69_	*ampC-1*	CC1	ST1	C
	7	1.5	+	*bla*_OXA−317_	*bla*_ADC−88_	Singleton	ST136	C
IMP, CAZ, SXT, GM, CIP	7	1.5	+	*bla*_OXA−317_	*bla*_ADC−88_	Singleton	ST136	B
	3	1.5	+	*bla*_OXA−317_	*bla*_ADC−88_	Singleton	ST136	C
	2	1.5	+	*bla*_OXA−90_	*ampC-39*	Singleton	ST78	C
	1	1.5	+	*bla*_OXA−69_	*ampC-1*	CC1	ST1	C
	1	1.5	+	*bla*_OXA−317_	*bla*_ADC−88_	Singleton	ST136	D
	1	1.5	+	*bla*_OXA−64_	*ampC-25*	CC25	ST25	B
	1	1.5	+	*bla*_OXA−64_	*ampC-25*	CC25	ST25	Singleton
	1	1.5	+	*bla*_OXA−90_	*ampC-39*	Singleton	ST78	C
IMP, CAZ, SXT, GM, CIP, SAM	1	1	-	*bla*_OXA−317_	*bla*_ADC−88_	Singleton	ST136	C
	1	1.5	+	*bla*_OXA−317_	*bla*_ADC−88_	Singleton	ST136	C
IMP, CAZ, SXT, GM, CIP, DXT, SAM	1	1.5	-	*bla*_OXA−317_	*bla*_ADC−88_	Singleton	ST136	B
IMP, CAZ, SXT, GM, CIP, DXT, MN	1	1.5	+	*bla*_OXA−317_	*bla*_ADC−88_	Singleton	ST136	C
IMP, CAZ, SXT, GM, CIP, DXT, MN, SAM	1	1.5	+	*bla*_OXA−69_	*ampC-1*	CC1	ST1	B
	1	1.5	+	*bla*_OXA−317_	*bla*_ADC−88_	Singleton	ST136	C
	1	1.5	-	*bla*_OXA−317_	*bla*_ADC−88_	Singleton	ST136	C

MIC, minimum inhibitory concentration; MLST, multilocus sequence typing; ST, sequence type; CC, clonal complex; RAPD, random amplified polymorphic DNA; IMP, imipenem, CAZ, ceftazidime; SXT, trimethoprim/sulphamethoxazole; GM, gentamicin; CIP, ciprofloxacin; DXT, doxycycline; MN, minocycline; SAM, ampicillin-sulbactam

### RAPD PCR results

**Fig. 1:**
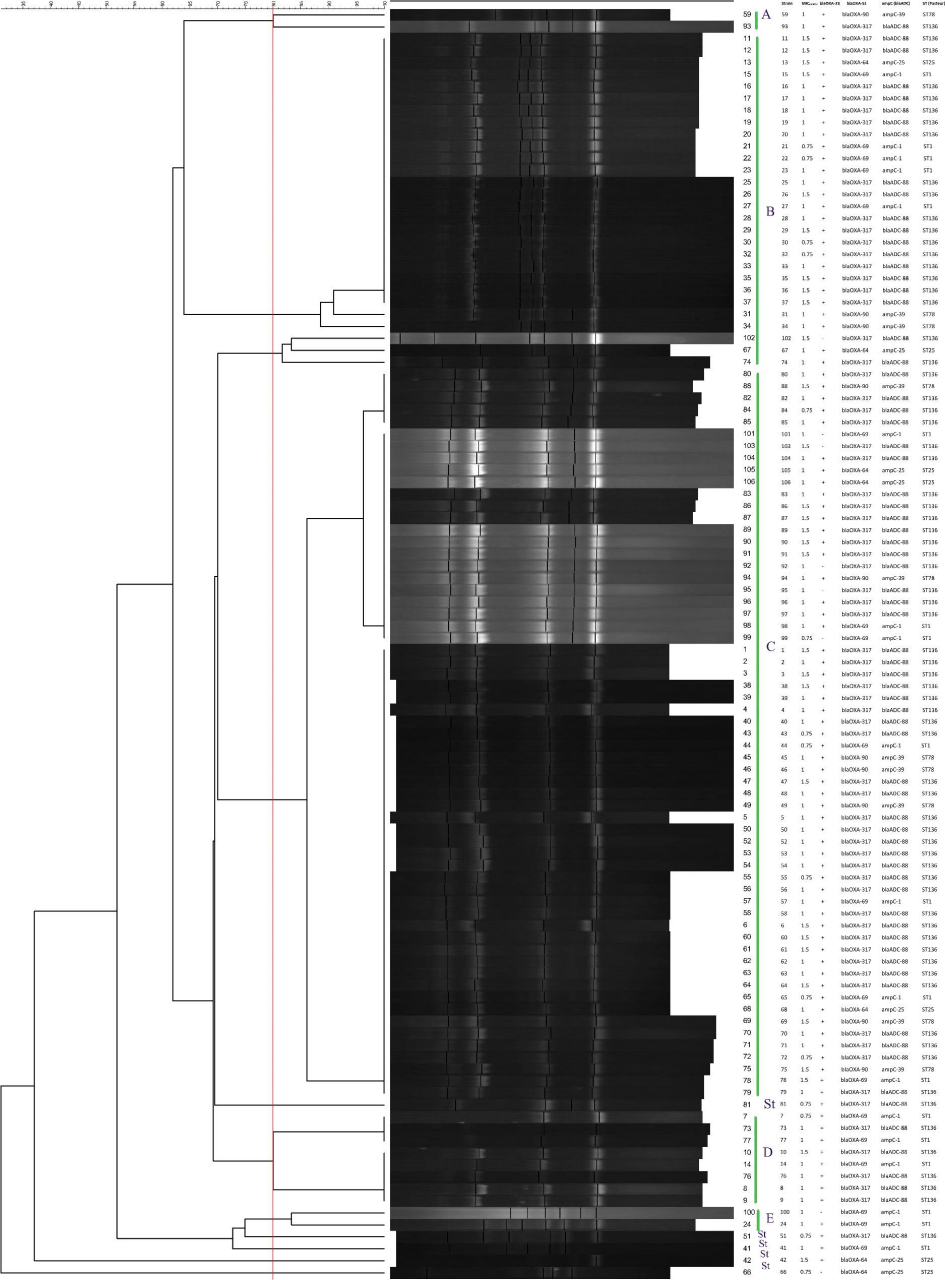
Random amplified polymorphic DNA (RAPD) analysis of 106 multi-drug resistant (MDR) *A. baumannii* isolated from the clinical setting. Clustering was performed using the unweighted pair group method with arithmetic averages (UPGMA). The vertical red line indicates the 80% similarity cut-off value. St., Singleton

RAPD analysis using the DAF4 primer with a similarity cut-off value of ≥ 80% revealed that the 106 *A. baumannii* isolates belonged to five RAPD types including A, B, C, D, and E. The majority of the *A. baumannii* strains were clustered in the RAPD type C including 61 (57.5%) strains and RAPD type B including 28 (26.4%) strains. However, the RAPD types A and E each contained 2 (1.9%) of *A. baumannii* strains, and were identified as the smallest clusters. In addition, 8 (7.5%) *A. baumannii* strains were grouped in RAPD type D and 5 (4.7%) strains were singleton. The dendrogram based on RAPD fingerprinting shows the obtained clusters. See additional information in Fig. 1.

### Multilocus sequence typing profile

**Fig. 2:**
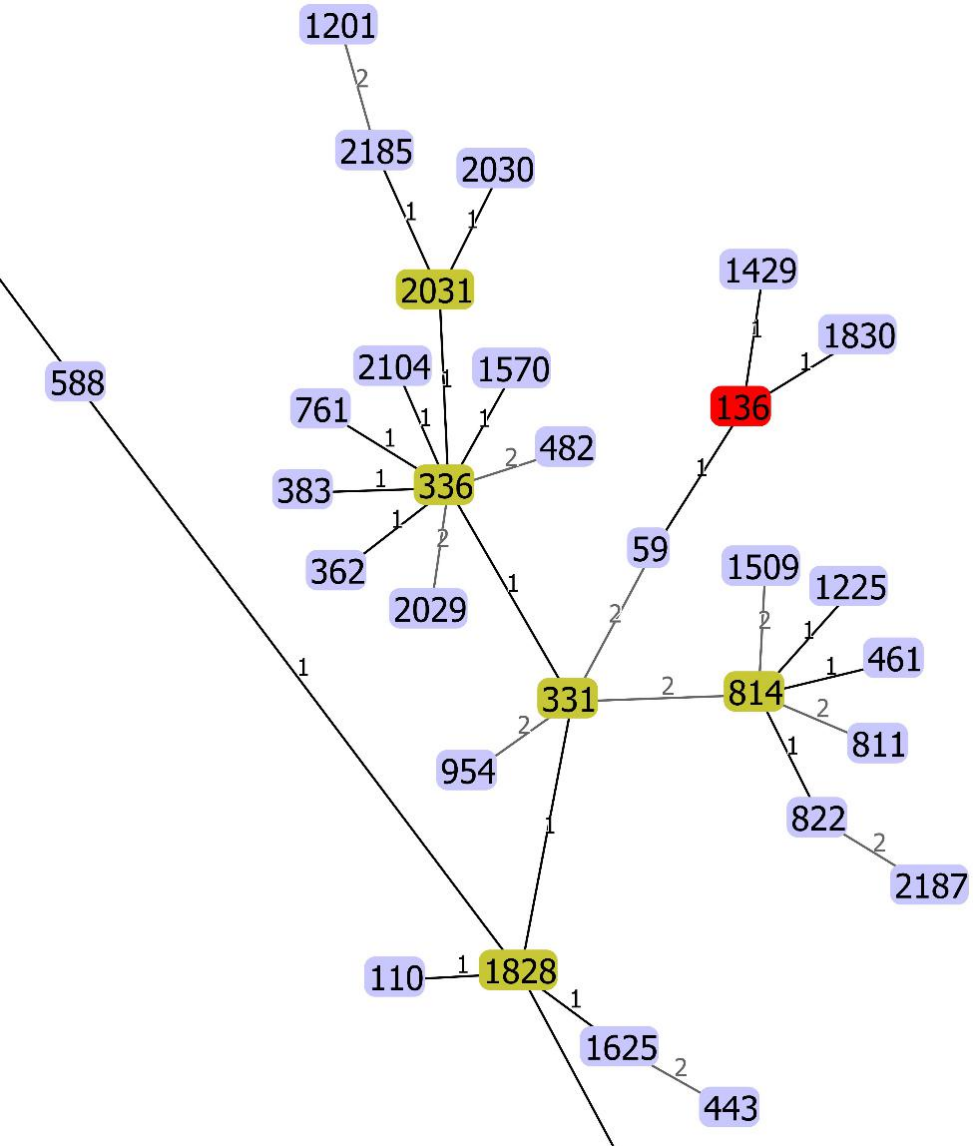
Minimum spanning tree (MST) of the sequence types associated with ST136 (Pasteur scheme). The difference allele number of two STs has been shown on the black lines. Clonal relatedness of ST136 (red rectangle) shows, this ST is closely related to ST59, ST1429 and ST1830.

MLST analysis detected four ST types including ST136, ST1, ST25 and ST78 in 71 (67.0%), 18 (17.0%), 7 (6.6%) and 10 (9.4%) of *A. baumannii* strains, respectively. The allele numbers for *cpn60*, *fusA*, *gltA*, *pyrG*, *recA*, *rplB* and *rpoB* (Pasteur scheme) in different sequence types are as follows: ST136 (3, 2, 19, 25, 5, 2, and 5), ST1 (1, 1, 1, 1, 5, 1, and 1), ST25 (3, 3, 2, 4, 7, 2, and 4), ST78 (25, 3, 6, 2, 28, 1, and 29). The clonal relatedness of ST136 (Pasteur scheme) associated with other STs has been shown in Fig. 2.

## Discussion

Severe infections caused by *A. baumannii* in burn patients, due to the increasing resistance to all or most of the available antibiotics, including carbapenems, have been a major concern in the last decade [2]. The apparent dominance of several multidrug-resistant lineages around the world highlights the importance of elucidating the distribution of *A. baumannii* isolates not only globally and nationally, but also in individual hospitals [9]. ST2 (Pasteur scheme) is the most widespread clone on the planet [24]. This ST frequently appears in CRAB and MDR clinical isolates [25]. However, in this cross-sectional study, we did not find ST2 among the clinical isolates. Multiple antimicrobial resistance may provide a selective advantage and contribute to the clonal expansion. It should be noted that a significant number of isolates belonged to ST1 (CC1) and 7 isolates also represented ST25 (CC25), both of them are multi-resistant clones with global distribution [26, 27]. The RAPD clustering results indicated that each ST type contained heterogeneous PAPD profiles. The high prevalence of divergent strains of STs identified by RAPD-PCR method, which belong to global STs such as ST1, ST25 in the clinical setting. It indicates the sustainable adaptation of these lineages to various environments. According to the available evidence, the sequence typing of *bla*_OXA−51_-like and *ampC* yields informative results that are broadly consistent with those obtained by MLST [11, 13]. However, the RAPD results did not fully match the MLST data. It seems that the dual-sequence typing method could be considered an accurate, fast and non-expensive procedure instead of MLST in developing countries.

ST78 as a globally distributed clone has been detected among carbapenem resistance isolates and appears along with international clonal lineages I-III and ST25 [28, 29]. It appears that the co-occurrence of heterogeneous STs depends on spatial and temporal conditions. The amount of antibiotic selection pressure, entry and circulation of different lineages and change of hostile conditions can change the population structure of isolates in clinical settings.

The resistance patterns of the strains highlighted that although all strains were absolutely MDR, all of them were sensitive to colistin. Nevertheless, 26 (24.5%) of the strains were diagnosed with colistin MIC of 1.5 µg/ml. Studies have shown that in clinical settings, colistin-resistant *A. baumannii* strains can emerge from colistin-sensitive strains by acquiring new features, in addition increasing use of the colistin, coupled with clonal expansion can amplify this trend [30]. Considering that colistin is used as a last resort therapeutic option in combination with antibiotics such as ampicillin-sulbactam or aminoglycosides in the treatment of infections caused by highly resistant *A. baumannii* strains. These results are noteworthy and the authors think that clinical isolates are on brink of colistin resistance due to continuous antibiotic selective pressure in the clinical setting.

Consistent with many studies, *bla*_OXA−23_-like gene was the most frequent gene which was identified in 92.5% of *A. baumannii* isolates [3, 5, 31]. A high prevalence of *bla*_OXA−23_-like gene has been reported in many regions of the world in carbapenem-resistant *A. baumannii* strains and presumably has gradually replaced with OXA-58 oxacillinases, which has been widespread in the past [3, 32]. Evidently, the carbapenem resistance in the present study, in accordance with other studies in Iran, is mainly to be driven by metallo-beta-lactamases, efflux pumps and CHDLs (*e.g. bla*_OXA−23_-like) [21, 33]. Many reports have shown that the linkage of IS*Aba1*/*bla*_OXA−23_-like and over-expression of *bla*_OXA−23_ gene is probably one of the causes of carbapenem resistance in *A. baumannii* isolates [34, 35].

The results obtained from typing of *A. baumannii* strains using the mentioned methods revealed that there was a high prevalence (67.0%, 71/106) of ST136 among MDR *A. baumannii* strains with sequence-based *bla*_OXA−51_-like allele type *bla*_OXA−317_. ST136^Pas^ has been reported from Taiwan and Kurdistan Region, Iraq [36, 37]. The strains of this ST were carbapenem-resistant and harbored *bla*_OXA−23_-like. They were mainly isolated from the burn wound at the burn units of hospitals. In addition, our analysis from PubMLST database showed that the ST136 (Pasteur scheme) corresponds to ST460 (Oxford scheme). PubMLST database has reported it from Taiwan (TTMHH-E9 strain, isolated from the environment, 2010), Iraq (IS-116 strain, isolated from wound), and the USA (OIFC065 strain, 2003). The genome analysis of IS-116 and OIFC065 strains demonstrated that they harbored *bla*_OXA−317_ and *bla*_ADC−88_ which confirmed our data.

In the present study, the typing of *A. baumannii* clinical isolates was conducted by methods including rapid and cost-effective allele typing of *bla*_*OXA−51*_-like and *ampC* and RAPD-PCR methods. However, the emergence of unrelated singleton clones has complicated the understanding of the population structure of *A. baumannii*, and the use of more precise methods such as whole genome sequencing (WGS) to demonstrate the evolutionary relatedness of circulating clones in clinical settings can be beneficial.

## Conclusion

A high prevalence of *bla*_OXA−23_-forming carbapenem-resistant *A. baumannii* (CRAB) was detected, belonging to the widely distributed ST136^Pas^ (singleton) in the clinical setting. Moreover, *bla*_OXA−23_-like MDR clones with global spread, including ST1, and emerging lineages (e.g., ST25 and ST78) have also been identified. Interestingly, there was no trace of ST2, the most widespread clone worldwide, in this clinical setting. It appears that the heterogeneous population structure of *A. baumannii* is due to spatial and temporal conditions. In addition, the dual-sequence typing method (*bla*_OXA−51_-like and *ampC* typing) could be considered as an accurate, fast, and not costly procedure instead of MLST in places with low financial resources.

## Data Availability

All data analyzed during this study are included here and available at GenBank database (https://www.ncbi.nlm.nih.gov/genbank/) and PubMLST (https://pubmlst.org/).
